# Persistence of major socio-economic inequalities in childhood measles–mumps–rubella vaccination coverage and timeliness under vaccination mandates, France, 2015 to 2024

**DOI:** 10.2807/1560-7917.ES.2025.30.16.2400674

**Published:** 2025-04-24

**Authors:** Dimitri Scronias, Lisa Fressard, Laure Fonteneau, Valérie Guagliardo, Pierre Verger

**Affiliations:** 1 ORS PACA, Southeastern Health Regional Observatory, Marseille, France; 2 Santé publique France (French National Public Health Agency), Saint-Maurice, France; 3 Unité des Virus Émergents (UVE: Aix-Marseille Univ, Università di Corsica, IRD 190, Inserm 1207, IRBA), Marseille, France

**Keywords:** vaccination coverage, vaccination timeliness, MMR vaccine, measles, equality

## Abstract

**Introduction:**

Since the late 2000s, several major measles epidemics have occurred in Europe, including France. In 2017, the French Health Ministry extended from three to 11 the number of mandatory childhood vaccines required for preschool and primary school admission; these included the vaccine against measles, mumps and rubella (MMR).

**Aim:**

Our aim was to assess if this measure helped to improve MMR vaccine timeliness (VT) or reduce socioeconomic inequalities in MMR vaccine coverage.

**Methods:**

A nationwide study of three birth cohorts (2015, 2017, 2019) followed up 2.1 million children for 48 months to assess the course of the timeliness of MMR vaccine dispensation, before and after it became mandatory in France (January 2018). Data came from the French national health insurance fund drug reimbursement database.

**Results:**

Despite improvements from 2015 to 2019, pharmacies dispensed MMR vaccines late for 33% of children in the 2019 cohort (mean cumulative delay compared with recommended dates: 7.1 months). Vaccines for children from low-income families were dispensed later (mean delay of at least +1 month) than those from higher-income families. The 2019 cohort did not reach the 95% WHO target of two MMR doses at 24 months of age, nor at 48 months.

**Discussion:**

With measles intensifying worldwide, these vaccination delays and inequalities may contribute to the resurgence of epidemics. In addition to vaccination mandates, an ambitious public health policy is needed to reduce inequalities in access to vaccination and to improve parents’ vaccine acceptance through educational strategies.

**Key public health message**

**What did you want to address in this study and why?**

In the context of major measles epidemics and suboptimal vaccine take-up, the French Health Ministry extended in 2017 the number of mandatory childhood vaccines from three to 11. We assessed if these mandates helped to improve the time between the recommended vaccination date and the actual vaccination for the measles–mumps–rubella (MMR) vaccine and reduce socioeconomic inequalities in this vaccination timeliness.

**What have we learnt from this study?**

Despite improvements in timeliness after the implementation of the mandates, vaccination delays are still substantial: a third of the children born in 2019 did not have their MMR vaccine dispensed on time (average delay of 7 months, or 8 months among those in a situation of socioeconomic deprivation), and the World Health Organisation’s target of 95% vaccination coverage for the MMR vaccine was still not reached 24 months after birth.

**What are the implications of your findings for public health?**

Long vaccination delays contribute to the resurgence of epidemics, and this risk is particularly relevant among socio-economically disadvantaged populations, who face multiple barriers in the access to care and prevention. In conjunction with vaccination mandates and strategies to increase parents’ vaccine acceptance, public health policies to improve equity in immunisation are essential to reduce the risks of new measles epidemics.

## Introduction

A major measles epidemic in Europe between 2008 and 2012 resulted in more than 23,000 cases in France — more than half the European total [1]. In 2013, the French Health Ministry modified its measles vaccination recommendations. It set a target level for vaccination coverage (VC) at age 24 months of at least 95% for the first dose and at least 80% for the second, with the recommended date of the first dose at 12 months and the second at 16–18 months, consistent with the World Health Organization‘s (WHO) recommendations [2]. Since vaccine acceptance in France was low among 40% of parents of young children [3], the Ministry launched in 2016 a broad consultation of the general public and healthcare professionals (HCPs) on how to improve childhood vaccination coverage [4]. It then extended the number of mandatory childhood vaccines required for admission to preschools and primary schools from three (diphtheria-tetanus-polio) to 11, including measles–mumps–rubella (MMR) [5], to help France reach the WHO's goal: 95% coverage for the vaccines recommended in each country. The law, enacted on 27 October 2017, took effect on 1 January 2018. 

In terms of recommended timing, besides the epidemiological reasons [2], the French MMR vaccination schedule (especially the second dose at 16–18 months) was adopted because of the high proportions of 2-year-olds (40% [6]) and 3-year-olds (≥ 97% by 2021 [7]) in preschools. Several European neighbouring countries, e.g. Germany, Monaco, and Switzerland, have an MMR vaccination schedule similar to that of France; others vary, with a second dose recommended much later (e.g. age of 5–6 years in Italy, 3 years in the United Kingdom) [8]. 

Assessments of the impact of these new mandates indicate that VC rates have improved for several childhood vaccines, including MMR [5,9]. Two important questions about their effects nonetheless remain. The first is whether or not they have improved vaccine timeliness (VT) (i.e. adherence to the officially recommended dates [10]) and achieved the 95% coverage target for both MMR doses before 24 months of age. Timeliness is essential because late vaccination facilitates virus circulation [10,11], particularly of highly contagious viruses such as measles. The second question is if inequalities in childhood VC and VT by family socioeconomic status have diminished. These persist in high-income countries [12] and even worsened in some places during the COVID-19 pandemic [13]. Equity in immunisation is one of the main strategic pillars of the WHO 2030 Immunization Agenda.

This article aims to provide answers to these two questions for measles vaccination among children covered by the French national health insurance fund (NHIF) with information from its drug reimbursement database. Because this database cannot reliably track some underserved communities and minorities (e.g. irregular migrants, individuals without housing), this article cannot cover those.

## Methods

### Population

We constructed three birth cohorts and followed them up from birth to age 48 months: one cohort well before the mandates' implementation (2015), one shortly before (2017) and one afterwards (2019). In France, vaccinations for 90% of children are prescribed by general practitioners or paediatricians; community pharmacists then dispense the vaccine to a parent, simultaneously recording this delivery in the NHIF data system (NHDS). However, mother–child protection centres (MCPCs) keep vaccines in stock and administer them, vaccinating less than 10% of all children free of charge. The NHDS does not record this vaccine delivery. We excluded children potentially vaccinated at MCPCs from our analyses by the application of a previously published methodology [5,9]. Further details on this methodology are appended in Supplement 1.

### Data collection

The NHDS data extraction took place in March 2024. To estimate VC and VT, we retrieved the dates (month, year) when pharmacies dispensed the MMR vaccine. We also retrieved an individual-level poverty indicator, the only one available in the NHDS: coverage of children's families by NHIF-subsidised social health insurance (SSHI: yes/no), which provides low-income families (i.e. those below the poverty line) with free access to healthcare based on financial criteria; it is a reliable indicator of low-income people in France. In 2019, the annual income level below which a three-person household could receive SSHI was EUR 16,112 [14]. In addition, we used the French deprivation index, an ecological index measuring the average deprivation level of families by municipality of residence, for each child [15]. It is built from the median wage and the following proportions: secondary school graduates among the population older than 15 years, blue-collar workers in the labour market, and unemployed workers. Finally, we collected the number of medical consultations for each child during the study period (proxy indicator of their health status) and the size of the family's municipality of residence (to distinguish rural from urban areas).

### Measles–mumps–rubella vaccination coverage and delay estimations

We defined VC, for each MMR dose and each cohort, as the proportion of children in the cohort for whom this vaccine was dispensed (i.e. purchased) over a given period.

We used the vaccine dispensing date as a proxy for the injection date because the latter is not always recorded in the NHDS. Because the anonymised database contains only the month and year of birth, MMR vaccine doses were considered late if they were delayed more than 2 months past the recommended dates (from month 14 for the first dose and month 20 for the second, see Supplement 2 for more details on delay estimations). Children who had not received any MMR dose by age 48 months were excluded from the VT descriptive statistics.

### Statistical analyses

We described the course of MMR VC over time from the age of 6 months, the percentage of children late for their vaccinations and the corresponding average delays (in months) for each of the three birth cohorts and for each (first and second) MMR dose — all before and after stratification for the poverty indicator (SSHI).

To analyse the effects of socioeconomic deprivation (at the individual and municipality levels) on vaccination delays by birth cohort, we implemented two log-normal accelerated failure time (AFT) models among children vaccinated late for each MMR dose. This approach allowed us to model children’s vaccination as a function of time, estimate the association between the moment of vaccination and several explanatory variables, and take unvaccinated children (right-censoring) into account in the estimates [16]. We controlled for birth cohort, sex, number of medical consultations for each child over the study period, size of the family's municipality of residence and, only for modelling the second dose, the length of the first dose's delay (in number of months; a timely first dose had a value of 0).

### Sensitivity analyses

To assess the impact of excluding children potentially vaccinated in MCPCs from our analyses, we included them in the 2019 cohort, imputing to them high MMR VC rates (98% for dose 1, and 95% for dose 2) and shorter delays (half the late vaccination rate and average delays in the main analysis) assuming that these families might have had better access to MCPCs than to general practitioners and paediatricians.

All analyses were performed with R 4.1.2.

## Results

Overall, 725,079 of 830,585 (87.3%) children were included in the 2015 birth cohort, 704,768 of 788,769 (89.4%) in the 2017 cohort, and 678,773 of 751,066 (90.4%) for 2019. The percentage of children excluded from the analyses because potentially vaccinated in MCPCs was higher among low-income than higher-income families. Supplementary Table S1 provides more details on numbers of children excluded, by SSHI status.

**Figure 1:**
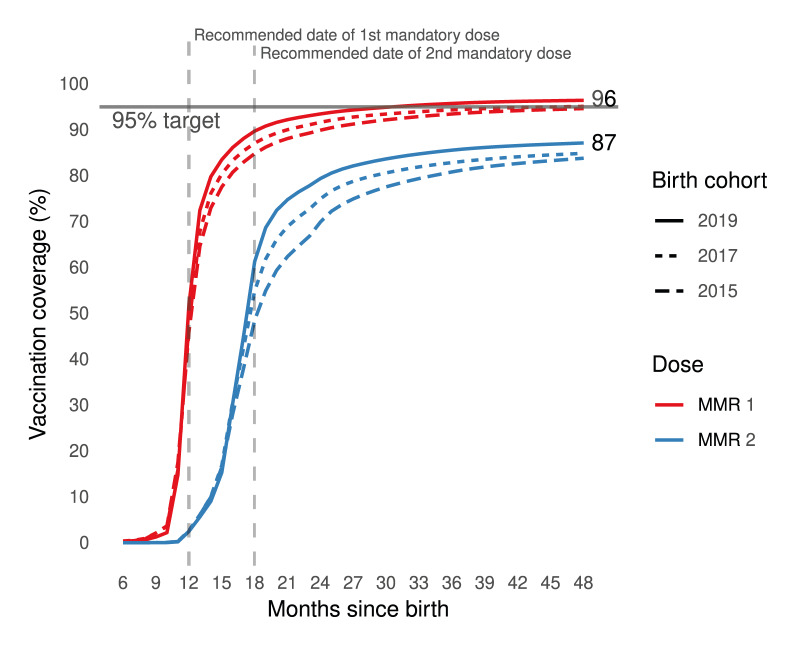
Dynamics of trends in measles–mumps–rubella vaccine coverage^a^, among three birth cohorts^b^, France (n = 2,108,620)

At 48 months of age, VC for MMR dose 1 rose from 94.6% in the 2015 cohort to 96.4% in the 2019 cohort, and VC for full vaccination (two doses) increased from 83.8% to 87.1% (Table 1); the proportion of children receiving only one dose decreased from 10.8% to 9.3%. The 2017 cohort, although not subject to the new law, had a slightly higher full MMR VC than the 2015 cohort. In the 2019 cohort, VC for MMR dose 2 at age 24 months — 79.4% — did not reach the 95% target (Figure 1).

**Table 1:** Measles–mumps–rubella vaccination coverage^a^, percentage of children vaccinated late and mean delays at age 48 months, in each birth cohort, France (n = 2,108,620)

Variable	Denominator	Cohort birth year^b^		
		2015	2017	2019
		n = 725,079	n = 704,768	n = 678,773
**Proportion in %**				
First MMR vaccine dispensed	All children	94.61	95.09	96.41
Late^c^ first dose	Children with at least one dose dispensed	31.72	28.62	24.85
Second MMR vaccine dispensed	All children	83.80	84.86	87.13
Late second dose	Children with both doses dispensed	33.37	26.16	20.37
**Mean delay^d^ in months**				
First MMR dose	Children with at least one dose dispensed, late first dose	5.9	5.3	5.2
Second MMR dose	Children with at least one dose dispensed, late second dose	6.2	5.6	5.4
Cumulative^e^ doses	Children with at least one dose dispensed, late first and/or second doses	8.2	7.3	7.1

MMR: measles–mumps–rubella.

^a^ The date when pharmacies dispensed the MMR vaccines served as proxy for the injection date to estimate vaccination coverage rates.

^b^ This study included only infants born in 2015, 2017 and 2019, who were alive 4 years after birth and who had a diphtheria, tetanus and pertussis vaccine dispensed at least once (before 2018) or a pentavalent or standard hexavalent childhood vaccination dispensed at least once (2018 onwards) by their first birthday: this condition was applied to exclude children vaccinated in mother–child protection centres [5].

^c^ MMR vaccine doses were considered dispensed late from month 14 for the first dose and month 20 for the second dose onward. See Supplement 2 for the handling of specific cases, such as a first dose dispensed after the mandatory date for the second.

^d^ Children without an MMR vaccine dispensed by age 48 months were excluded from the delay calculation.

^e^ Mean cumulative delay includes children whose first and/or second MMR dose was dispensed late.

Data source: National health data system, March 2024.

MMR: measles–mumps–rubella.

^a^ The date when pharmacies dispensed the MMR vaccines served as proxy for the injection date to estimate vaccination coverage rates.

^b^ This study included only children born in 2015, 2017 and 2019, alive 4 years after birth and who had had a diphtheria, tetanus and pertussis vaccine dispensed at least once (before 2018) or a pentavalent or standard hexavalent childhood vaccination dispensed at least once (2018 onwards) by their first birthday: this condition was applied to exclude children vaccinated in mother–child protection centres [5]. Final numbers of children in the birth cohorts, after these exclusions: n (2015) = 725,079; n (2017) = 704,768; n (2019) = 678,773.

Data source: National health data system, March 2024.

### Measles–mumps–rubella vaccination coverage dynamics between the three birth cohorts

The improvement in the dynamics by which vaccines were dispensed between the 2015 and 2019 cohorts shows a reduction in average time to vaccination, more marked for dose 2 than dose 1 (Figure 1). The proportion of children whose first MMR dose was dispensed late decreased from 31.7% in the 2015 cohort to 24.9% in the 2019 cohort, and for the second MMR dose, from 33.4% to 20.4% respectively. The cumulative delay (estimated among those late for one or two doses (45.4% for 2015 and 32.5% for 2019) fell correspondingly from 8.2 to 7.1 months at age 48 months (Table 1). In Supplementary Table S4, we append the details of sensitivity analyses for the threshold to define a vaccination delay; these indicated that a 2-month delay underestimated percentages of children with untimely MMR vaccination. 

### Differences between children from different socioeconomic backgrounds

**Figure 2:**
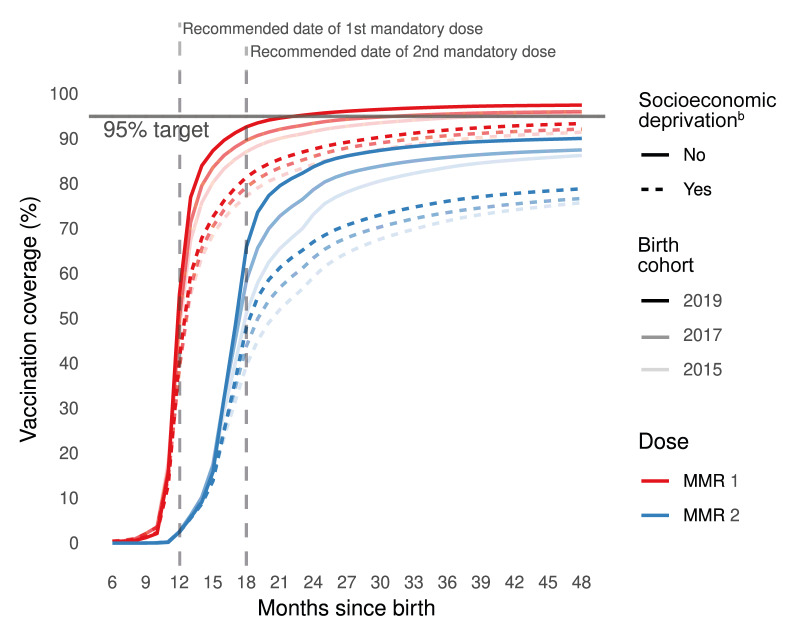
Dynamics of trends in measles–mumps–rubella vaccination coverage^a^, above and below the poverty line^b^, among three birth cohorts^c^, France (n = 2,108,620)

The dynamics of MMR vaccination differed between children from families below vs above the poverty line (the annual income level below which a household could receive SSHI) with MMR vaccine dispensed later and at lower rates among the low-income families (below the poverty line), for both doses (Figure 2). In the 2015 cohort, the proportion of children from low-income families late for the first MMR dose at age 48 months was +9.8 percentage points (ppts) higher than for more affluent children, while this difference rose to +14.9 ppts in the 2019 cohort (Table 2). Second-dose VC at age 24 months among the 2019 cohort reached 67.1% for children from low-income families, vs 83.8% for the others; at age 48 months, these figures were respectively 78.9% and 90.1%. By age 48 months, among vaccinated children to whom both doses were dispensed, the mean cumulative delay was longest among the children from low-income families: 9.2 months in the 2015 cohort (vs 7.8 months for those above the poverty line, Table 2), and 8.3 months in the 2019 cohort (vs 6.4 months). Across the three cohorts, the cumulative delay of dispensing for both doses fell more slowly in the low- than in the higher-income group. Consequently, the gap at age 48 months between the 2015 and 2019 cohorts widened by half a month (Table 2).

MMR: measles–mumps–rubella.

^a^ The date when pharmacies dispensed the MMR vaccines served as proxy for the injection date to estimate vaccination coverage rates.

^b^ Families were defined as below the poverty line if they were covered by subsidised social health insurance at any time during the study follow-up. Percentage of recipients in 2015: 23.7%; 2017: 24.6%; 2019: 26.3%.

^c^ This study included only children born in 2015, 2017 and 2019, who were alive 4 years after birth and who had a diphtheria, tetanus and pertussis vaccine dispensed at least once (before 2018) or a pentavalent or standard hexavalent childhood vaccination dispensed at least once (2018 onwards) by their first birthday: this condition was applied to exclude children vaccinated in mother–child protection centres [5]. Final numbers of children in the birth cohorts, after these exclusions: n (2015) = 725,079; n (2017) = 704,768; n (2019) = 678,773.

Data source: National health data system, March 2024.

**Table 2:** Measles–mumps–rubella vaccination coverage^a^, percentages of children vaccinated late and mean delays at age 48 months, above and below the poverty line^b^, France (n = 2,108,620)

Variable	Denominator	Cohort birth year^c^						Trends^d^ of differences between low-and higher-income, 2015 to 2019
		2015		2017		2019		
		Low income		Low income		Low income		
		No	Yes	No	Yes	No	Yes	
Percentage		*%*	∆ ppt^e^	%	∆ ppt^e^	%	∆ ppt^e^	ppt
First MMR vaccine dispensed	All children	95.6	−4.1	96.1	−3.9	97.5	−4.1	0.0
Late^f^ first dose	Children with at least one dose dispensed	29.5	+9.8	25.7	+12.3	21.1	+14.9	+5.1
Second MMR vaccine dispensed	All children	86.3	−10.5	87.5	−10.8	90.1	−11.2	+0.7
Late second dose	Children with both doses dispensed	31.9	+6.7	24.2	+8.8	17.7	+11.2	+4.5
Mean delay^g^ in months								
First MMR dose	Children with at least one dose dispensed, late first dose	5.51	+1.38	4.77	+1.58	4.54	+1.76	+0.4
Second MMR dose	Children with at least one dose dispensed, late second dose	6.05	+0.68	5.40	+0.81	5.14	+0.90	+0.2
Cumulative doses^h^	Children with at least one dose dispensed, late first and/or second doses	7.78	+1.44	6.75	+1.72	6.36	+1.97	+0.5

MMR: measles–mumps–rubella; ppt: percentage points.

^a^ The date when pharmacies dispensed the MMR vaccines served as proxy for the injection date to estimate vaccination coverage rates.

^b^ Families were defined as below the poverty line if they were covered by subsidised social health insurance at any time during the study follow-up. Percentage of recipients in 2015: 23.7%; 2017: 24.6%; 2019: 26.3%.

^c^ This study included only children born in 2015, 2017 and 2019, who were alive 4 years after birth and who had a diphtheria, tetanus and pertussis vaccine dispensed at least once (before 2018) or a pentavalent or standard hexavalent childhood vaccine dispensed at least once (2018 onwards) by their first birthday: this condition was applied to exclude children vaccinated in mother–child protection centres [5]. Final numbers of children in the birth cohorts, after these exclusions: n (2015) = 725,079; n (2017) = 704,768; n (2019) = 678,773.

^d^ Formula:|X_2019, deprived_ - X_2019, non-deprived_| - |X_2015, deprived_ - X_2015, non-deprived_|, where X is one of the indicators in the rows of the table and |X| the absolute value of X. For each variable, this describes the trend of the difference in percentage points between children from low-income families and other children from 2015 to 2019.

^e^ Difference, in ppt, between children who were living below the poverty line and children who were not.

^f^ MMR vaccine doses were considered dispensed late from month 14 for the first dose and month 20 for the second dose onward. See Supplement 2 for the handling of specific cases, such as a first dose dispensed after the mandatory date for the second.

^g^ Children who had not received the dose of vaccine by age 48 months were excluded from the calculation.

^h^ Mean cumulative delay includes children whose first and/or second MMR dose was dispensed late.

Data source: National health data system, March 2024.

Additional sensitivity analyses are provided in Supplementary Table S3; these showed that including children vaccinated in MCPCs in our analyses had a limited impact on overall VC, even for our most optimistic assumptions for vaccination in MCPCs: VC rose from 96.4% to 96.6% at age 48 months for the first dose and from 87.1% to 87.9% for the second. Differences were slightly higher — though limited — among children from low-income families, with VC increasing from 93.4% to 94.1% for dose 1, and from 78.9% to 81.3% for dose 2. Differences in vaccination delays were also small, with reductions after imputation not exceeding 0.3 months.

Finally, the AFT model for children vaccinated late (Table 3) found that time until dispensing of the first MMR dose was 52% longer among children from low-income families than among the others (33% for dose 2), all else being equal. Moreover, delays for dose 1 increased by 3% (2% for dose 2) for each unit increment of the deprivation index of the family's municipality of residence. Children from later birth cohorts had statistically significantly shorter delays for the first dose only (2017: −14%, 2019: −26%).

**Table 3:** Factors associated with delayed dispensing of measles–mumps–rubella vaccine for untimely vaccinated children, accelerated failure time model, three birth cohorts^a^, France (dose 1: n = 588,841; dose 2: n = 611,252)

Explanatory variable	Duration of MMR vaccine delay^b^	
	aTR	99.9% CI
Dose 1	n = 588,841^c^	
**Sex**		
Male	Reference	
Female	0.97	0.96–0.99
**Birth cohort**		
2015	Reference	
2017	0.86	0.85–0.87
2019	0.74	0.73–0.76
**Below the poverty line^d^**		
No	Reference	
Yes	1.52	1.50–1.54
Deprivation level in the municipality of residence^e^ Mean: 0.0 (range: −6.1–7.2)	1.03	1.03–1.04
Number of consultations with general practitioners or paediatricians Mean: 30 (range: 1–88)	0.98	0.98–0.98
**Type and size of municipality**		
Intermediate city between 5,000 and 200,000 inhabitants^f^	Reference	
Paris	1.08	1.06–1.10
Large suburbs (≥ 200,000 inhabitants)	0.98	0.96–1.00
Large city centres (≥ 200,000 inhabitants, except Paris)	1.05	1.02–1.07
Small cities or rural areas (< 5,000 inhabitants)	1.00	0.99–1.02
Dose 2	n = 611,252^g^	
Length of delay (in number of months) of vaccination for first dose Range: 0–35	1.06	1.06–1.06
**Sex**		
Male	Reference	
Female	1.00	0.99–1.01
**Birth cohort**		
2015	Reference	
2017	0.98	0.97–1.00
2019	0.99	0.97–1.01
**Below the poverty line^d^**		
No	Reference	
Yes	1.33	1.31–1.35
Deprivation level in the municipality of residence^e^ Mean: 0.0 (range: −6.1–7.2)	1.02	1.01–1.02
Number of consultations with general practitioners or paediatricians Mean: 30 (range: 1–88)	0.99	0.99–0.99
**Type and size of municipality**		
Intermediate city between 5,000 and 200,000 inhabitants^f^	Reference	
Paris	1.40	1.37–1.43
Large suburbs (≥ 200,000 inhabitants)	1.00	0.98–1.02
Large city centres (≥ 200,000 inhabitants, except Paris)	1.10	1.07–1.13
Small cities or rural areas (< 5,000 inhabitants)	0.96	0.94–0.98

aTR: adjusted time ratios; CI: confidence interval; MMR: measles–mumps–rubella.

^a^ Children born in 2015, 2017 and 2019, who were alive 4 years after birth and who had a diphtheria, tetanus and pertussis vaccine dispensed at least once (before 2018) or a pentavalent or standard hexavalent childhood vaccination dispensed at least once (2018 onwards) by their first birthday: this condition was applied to exclude children vaccinated in mother–child protection centres. Children with a very high number of consultations (> 88) over the study period or with missing data for covariates were excluded. Final numbers of children in the birth cohorts, after these exclusions: n (2015) = 653,263; n (2017) = 631,260; n (2019) = 607,205.

^b^ For the two MMR vaccine doses, vaccines were considered dispensed late from month 14 for the first dose and month 20 for the second dose onward. See Supplement 2 for the handling of specific cases, such as a first dose dispensed after the mandatory date for the second.

^c^ 1,299,887 children with a first dose dispensed on time were excluded.

^d^ Families were defined as below the poverty line if they were covered by subsidised social health insurance at any time during the study follow-up. 2015: 23.7%; 2017: 24.6%; 2019: 26.3%.

^e^ The indicator for ‘residence in a socially disadvantaged municipality’ was constructed at the level of municipalities, from four variables available from the French census: median wage by consumption units in the household, percentage of secondary-school graduates (passed baccalaureate exam) in the population older than 15 years, the percentage of blue-collar workers in the labour market, and the unemployment rate [5].

^f^ Classification from the National Institute of Statistics and Economic Studies (INSEE, 2020)

^g^ 1,277,476 children with a second dose dispensed on time or no first dose dispensed were excluded.

The accelerated failure time model estimates aTR associated with a category/value of an explanatory variable: a value greater than 1 indicates a longer delay until vaccination takes place, while a value less than 1 indicates a delay shorter than the reference category. For example, an aTR of 1.52 associated with low income means that time until the first MMR vaccine dose was dispensed was 52% longer among children from low-income families than among others.

Data source: National health data system, March 2024.

## Discussion

Our study shows that among the children vaccinated by HCPs in the community (ca 90% of children in France in 2019), estimated VC and VT for MMR doses 1 and 2 improved moderately after the new mandates came into effect in 2018. Nonetheless, VC for full MMR vaccination in the 2019 cohort remained clearly below the WHO 95% target, even at age 48 months, and one-third of these children had one or both MMR doses late, with a cumulative mean delay of 7 months. Moreover, children from low-income, compared with higher-income families, were less well protected against measles (coverage differences were substantial for dose 2, which may or may not have been administered after 48 months); their risk of delayed MMR vaccination was statistically significantly higher. Overall, our results indicate that the MMR vaccination mandates benefited low-income groups less than more affluent groups, and thus increased, rather than decreased, social inequalities in immunisation.

As 97% of children are enrolled in preschool at age 48 months and measles is highly contagious, inadequate enforcement of the new mandatory childhood vaccinations means that the law may not effectively prevent transmission should France experience another epidemic [17]. While the statute provides that children who are not fully vaccinated must be excluded from school, it is the preschool or nursery school that must ultimately take this complex and difficult-to-enforce decision and enforce it [18]. The worldwide epidemic risk is far from negligible. Measles has intensified since 2022, with major epidemics currently ongoing in South Asia and Africa [11,19] and a considerable rise in some parts of Europe [20]. Furthermore, international tourism from these continents to Europe had almost returned to pre-COVID-19 levels by 2023, exposing European countries to risks of measles importation and epidemic resurgence [11,21]. Vaccine coverage is not always adequate for collective protection in France or elsewhere in Europe [1,11,22], where measles vaccination delays are relatively common [10,23].

Risk of measles epidemics may be particularly high where under- or late-vaccinated population groups are over-represented. This is the case in 14 French départements (among 101 such French administrative divisions), concentrated in southern France and in overseas départements, where complete measles VC does not exceed 80% at the age of 33 months [9]. It is even more true in municipalities with high deprivation scores (most French cities with > 100,000 inhabitants).

We used the WHO Framework of Health System Components [24] to identify the important barriers that may affect MMR vaccination among low-income populations [25]: financial costs, the availability of services (regardless of cost), and the inadequate level of vaccine acceptance.

Firstly, financial costs remain a barrier for low-income and other marginalised groups to access vaccination. While the NHIF fully reimburses medical prescriptions for the MMR vaccine for children of families it insures, it pays only a portion of vaccine administration costs: those who cannot afford supplementary health insurance (ca 20% of the population in France) [26] may also be unable to pay these out-of-pocket costs. The complexity of the reimbursement system is a structural barrier contributing to high rates of non-utilisation of social benefits among low-income individuals often unable to navigate this system [27]. Since the beginning of the 2010s, reduced funding of the MCPCs has also impaired its long-standing ability to offer free vaccination to the general and especially the low-income population. Thus the population they serve and the number of consultations they offer have declined [28]. See also Supplementary Table S1 for trends in the number of children potentially cared for by MCPCs.

Secondly, the COVID-19 pandemic made access to healthcare services harder (making appointments online at vaccination centres, dematerialised vaccination certificates, teleconsultations, etc.) and revealed the dual divide — social and digital — separating groups including socially isolated or financially disadvantaged people, people with disabilities, and migrants from the rest of the population [29].

Furthermore, many European countries, especially France, are currently experiencing a severe scarcity of doctors [30], limiting the supply of time for consultations, especially for prevention. This scarcity is most marked in deprived areas and may partly explain the relation we observed between the increased risk of delayed MMR vaccination and the deprivation level of families' municipalities of residence.

Thirdly, lower vaccine acceptance in low-income and other underserved groups may contribute to insufficient and/or late MMR vaccination [25]. The monitoring of vaccine acceptance prevalence in France from 2000 through 2021 suggests the gap is widening between affluent and low-income populations, to the detriment of the latter [31]. A cross-sectional survey of ca 2,000 adults in France in 2023 showed that 12.4% of low-income participants were unfavourable towards MMR vaccination vs 7.6% among the higher-income participants (p < 10^−3^, personal communication: Fressard, July 2024). Trust in HCPs and the healthcare system are core determinants of vaccine acceptance. Among low-income individuals, previous experiences of care and discrimination (sex, gender, sexual preference, social class, race, age, disability) may undermine vaccine confidence [25] as may doubts about vaccine efficacy and safety potentially fuelled by various contextual and personal factors [32]: misinformation, inadequate health or overall literacy, some cultural or religious beliefs, adherence to conspiracy theories, etc. Socioeconomically vulnerable parents with little knowledge about childhood vaccination and mandates might be less likely than other parents to comply with mandatory vaccination [33].

Three primary categories of solutions are applicable to each of these factors: public policies promoting the removal of barriers; educational programmes for HCPs to help them improve their care of patients with complex needs; and interdisciplinary research programmes.

Policy and administrative steps to reduce barriers to vaccination (and healthcare) access for low-income groups include simplifying access to social rights, restoring MCPC funding, dealing with issues related to the longer consultation times (funding, task shifting, etc.) required to address the complex situations associated with socioeconomic deprivation, and ensuring that health services are provided in the mother tongue of minority populations and take their culture into account [25]. These steps could also help improve the confidence of low-income groups in both the healthcare system and HCPs. Outreach programmes with sustainable funding and trained professionals should be developed to reach isolated groups and accompany them to care.

Improved initial and continued training for HCPs is among the most useful steps possible to aid equity in immunisation by helping HCPs deal with the numerous difficulties of caring for low-income individuals, including communication difficulties linked to language skills, literacy and health literacy. A survey on caring for low-income patients among nearly 1,000 general practitioners in France in 2017 revealed that [34]: 75% perceived that prevention with these patients appeared ineffective; 54% did not feel adequately trained to deal with these patients, and nearly 50% did not feel adequately trained to adapt prevention messages to them. These results are consistent with the international literature [25] and underline the need for guidelines to help HCPs and improve the available training.

Priority areas for interdisciplinary research include understanding how low-income people cope with the complexity of the reimbursement system, the structural reasons for its persistence in the health system and how public policies could reduce it. Research is also needed to assess the specific needs, constraints, perceptions and opinions of the various disadvantaged groups based on their experiences of the healthcare system, and to better understand, more generally, the social, economic and cultural dynamics or logics of appropriation or rejection of health norms and how they influence the acceptance of coercive measures among socioeconomically vulnerable groups [35].

Findings show that personalised approaches based on emphatic listening, such as motivational interviewing, in different groups of this population have improved vaccine confidence and acceptance in some countries and may be especially appropriate with low-income groups in various cultural contexts [36,37]. Further research is needed to determine the conditions for their broader deployment.

Some limitations should be considered when interpreting our results. While they concern a large majority (90%) of children of the birth cohorts considered, caution is required in generalising them to the entire population of these birth cohorts because children potentially vaccinated against measles in MCPCs were excluded from our analyses [5] and because these children are more often from low-income. Nonetheless, among all low-income children in the 2019 cohort, only 15% were excluded because of potential vaccination in MCPCs; further details on the exclusions can be seen in Supplement 1. The method used to exclude children potentially vaccinated against measles in MCPCs [5] cannot exclude those whose first vaccine was dispensed in community pharmacies and their second later in a MCPC: it might lead to underestimating second dose VC and overestimating delays for the first dose. We append a sensitivity analysis in Supplementary Table S3, where assumption of high VC and low delay in children vaccinated in these centres only slightly modified our conclusions about VT and socioeconomic disparities. However, the substantial decline in the number of consultations available for children younger than 6 years in MCPCs since the 2010s suggests that these assumptions may be optimistic [28].

Our estimates of MMR VC are consistent with those already published [9]. Additional sensitivity analyses on ways to define vaccination delays are appended in Supplementary Table S4; they affected our results only marginally. Using the dates when MMR was dispensed in pharmacies as proxies for injection dates could have resulted in underestimating vaccination delays as injections could have occurred well after purchase, and in overestimating VC if vaccines were delivered but not administered. All in all, our sensitivity analyses indicate that our main conclusion remains valid: social inequalities in MMR vaccination coverage and delay persisted in the 2019 cohort despite the vaccine mandates.

As the database of the French National Health Insurance Fund does not allow access to the home addresses of insured persons, the municipality deprivation index cannot measure the heterogeneity within municipalities (except for Paris, Marseille and Lyon where it is available at the district level). Nonetheless, because it reflects — homogeneously across France — a major part of the spatial socioeconomic heterogeneity, its use is recommended for observing and analysing spatial health inequalities [15]. The type and size of municipality classification further improved the analysis of spatial health inequality by capturing effects not captured by the deprivation index. The share of people with low-income increases with municipality size and is, furthermore, higher in city centres than suburbs [38]. The impact of lockdowns on healthcare use during the COVID-19 pandemic in 2020, especially the first one from mid-March to the first week of May, might have affected the MMR vaccination dynamic [39]. However, our results for the 2019 cohort do not indicate any marked change over this period, and the curves for the cohort of children born in 2020 are the same, with the same gaps between children from low-income families and more affluent families (data not shown).

Finally, the inability to reliably track some specific underserved communities, such as irregular migrants, in the NHDS, prevented any specific analysis among these subpopulations within the category of low-income families.

Despite these various limitations, the NHDS covers almost the entire population of France and provides access to objective, reliable retrospective data, enabling us to study different birth cohorts.

## Conclusion

A moderate improvement in MMR VC and VT was observed for both doses after the extension of compulsory childhood vaccinations in 2018. However, the worsening of social inequalities in MMR vaccination following this extension highlights the need to try anticipating this kind of undesired effect before such public health policies are implemented. In-depth knowledge of the structural reasons why low-income groups may respond less than more affluent ones to changes in public health policy remains essential. Beyond this question, it is also important to understand the possible structural obstacles to anticipating this type of problem when drawing up vaccination policies. Addressing these research questions is essential to be better prepared to prevent and manage future epidemics. Finally, maintaining or improving trust in all the components of our societies in the healthcare system, its actors and vaccination remains a priority to accompany the coercive vaccination policies: this is a complex task requiring substantial resources to develop comprehensive, multicomponent interventions tailored to each specific context and to evaluate, deploy and follow them up.

## Supplementary Data

305000 Supplement

## References

[1] European Centre for Disease Prevention and Control (ECDC). European monthly measles monitoring (EMMO). Issue 8. Stockholm: ECDC; 2012. Available from: https://www.ecdc.europa.eu/sites/default/files/media/en/publications/Publications/SUR_EMMO_European-monthly-measles-monitoring-February-2012.pdf

[2] Santé publique France (SPF). Le Calendrier des vaccinations et les recommandations vaccinales 2013 selon l’avis du Haut Conseil de la santé publique. [The 2013 vaccination schedule and vaccination recommendations according to the advice of the High Council for Public Health]. Bull Epidemiol Hebd (Paris). 2013;14-15. French. Available from: https://www.hcsp.fr/Explore.cgi/Telecharger?NomFichier=hcsp_beh_14_15_2013.pdf

[3] Rey D Fressard L Cortaredona S Bocquier A Gautier A Peretti-Watel P Vaccine hesitancy in the French population in 2016, and its association with vaccine uptake and perceived vaccine risk-benefit balance. Euro Surveill. 2018;23(17):17-00816. 10.2807/1560-7917.ES.2018.23.17.17-00816 29717693 PMC5930729

[4] Ward JK Cafiero F Fretigny R Colgrove J Seror V . France’s citizen consultation on vaccination and the challenges of participatory democracy in health. Soc Sci Med. 2019;220:73-80. 10.1016/j.socscimed.2018.10.032 30408684

[5] Lévy-Bruhl D Fonteneau L Vaux S Barret AS Antona D Bonmarin I Assessment of the impact of the extension of vaccination mandates on vaccine coverage after 1 year, France, 2019. Euro Surveill. 2019;24(26):1900301. 10.2807/1560-7917.ES.2019.24.26.1900301 31266592 PMC6607743

[6] Caenen Y, Virot P. La part des enfants de moins de 3 ans confiés principalement à une assistante maternelle ou une crèche a presque doublé entre 2002 et 2021. [The proportion of children under 3 years old entrusted mainly to a childminder or a crèche almost doubled between 2002 and 2021]. Report no. 1257. Paris: Direction de la Recherche, des Études, de l'Évaluation et des Statistiques; 2023. French. Available from: https://drees.solidarites-sante.gouv.fr/sites/default/files/2023-02/ER1257MAJ.pdf

[7] Institut National de la Statistique et des Etudes Economiques (INSEE). Taux de scolarisation par âge. Données annuelles de 2000 à 2021. [School enrolment rate by age. Annual data from 2000 to 2021]. Montrouge: INSEE; 2023. Available from: https://www.insee.fr/fr/statistiques/2383587#tableau-figure1

[8] World Health Organization (WHO). Vaccination schedule for Measles. Geneva: WHO; [Accessed: 30 Jul 2024]. Available from: https://immunizationdata.who.int/global/wiise-detail-page/vaccination-schedule-for-measles?ISO_3_CODE=&TARGETPOP_GENERAL=&CODE=EURO

[9] Santé publique France (SPF). Bulletin de santé publique vaccination. Avril 2023. [Public Health Vaccination Bulletin. April 2023]. Saint-Maurice: SPF; 2023. French. Available from: https://www.santepubliquefrance.fr/determinants-de-sante/vaccination/documents/bulletin-national/bulletin-de-sante-publique-vaccination.-avril-2023

[10] Riise ØR Laake I Bergsaker MAR Nøkleby H Haugen IL Storsæter J . Monitoring of timely and delayed vaccinations: a nation-wide registry-based study of Norwegian children aged < 2 years. BMC Pediatr. 2015;15(1):180. 10.1186/s12887-015-0487-4 26563381 PMC4643514

[11] UK Health Security Agency (UKHSA). Risk assessment for measles resurgence in the UK. London: UKHSA; 2023. Available from: https://assets.publishing.service.gov.uk/government/uploads/system/uploads/attachment_data/file/1170146/risk-assessment-for-measles-resurgence-in-the-UK-2023.pdf

[12] Bocquier A Ward J Raude J Peretti-Watel P Verger P . Socioeconomic differences in childhood vaccination in developed countries: a systematic review of quantitative studies. Expert Rev Vaccines. 2017;16(11):1107-18. 10.1080/14760584.2017.1381020 28914112

[13] Spencer N Markham W Johnson S Arpin E Nathawad R Gunnlaugsson G The impact of COVID-19 pandemic on inequity in routine childhood vaccination coverage: a systematic review. Vaccines (Basel). 2022;10(7):1013. 10.3390/vaccines10071013 35891177 PMC9321080

[14] Caisse Nationale de l’Assurance Maladie (CNAM). Circulaire CIR-33/2019. Paris: CNAM; 2019. Available from: https://circulaires.ameli.fr/sites/default/files/directives/cir/2019/CIR-33-2019.PDF

[15] Rey G Jougla E Fouillet A Hémon D . Ecological association between a deprivation index and mortality in France over the period 1997 - 2001: variations with spatial scale, degree of urbanicity, age, gender and cause of death. BMC Public Health. 2009;9(1):33. 10.1186/1471-2458-9-33 19161613 PMC2637240

[16] Moore DF. Applied survival analysis using R. Chapter 10.3.7. Using the Weibull distribution to model survival data with multiple covariates. (Use R!). Cham: Springer International Publishing; 2016. Available from: http://link.springer.com/10.1007/978-3-319-31245-3

[17] World Health Organization (WHO). Immediate and targeted catch-up vaccination needed to avert measles resurgence. Geneva: WHO; 2023. Available from: https://www.who.int/europe/news/item/10-02-2023-immediate-and-targeted-catch-up-vaccination-needed-to-avert-measles-resurgence

[18] Perrot N. Obligation vaccinale pour les élèves. [Mandatory vaccination for students]. Paris: Syndicat National des lycées, collèges, écoles et du supérieur. 2024. French. Available from: https://snalc.fr/obligation-vaccinale-pour-les-eleves

[19] World Health Organization (WHO). Measles reported cases and incidence. Geneva: WHO. [Accessed: 12 Dec 2024]. Available from: https://immunizationdata.who.int/global/wiise-detail-page/measles-reported-cases-and-incidence?GROUP=WHO_REGIONS&YEAR=

[20] European Centre for Disease Prevention and Control (ECDC). Measles on the rise again in Europe: time to check your vaccination status. Stockholm: ECDC; 2025. Available from: https://www.ecdc.europa.eu/en/news-events/measles-rise-again-europe-time-check-your-vaccination-status

[21] Eurostat. Nights spent at tourist accommodation establishments by country of origin of the tourist. Africa, Asia. Luxembourg: Eurostat. [Accessed: 12 Dec 2024]. Available from: https://ec.europa.eu/eurostat/databrowser/view/tour_occ_ninraw__custom_14426359/bookmark/line?lang=en&bookmarkId=3e283b5c-f24f-419e-a337-a89a1c82261b

[22] World Health Organization (WHO). Measles – European Region. Geneva: WHO; 2019. Available from: https://www.who.int/emergencies/disease-outbreak-news/item/2019-DON140

[23] Haider EA Willocks LJ Anderson N . Identifying inequalities in childhood immunisation uptake and timeliness in southeast Scotland, 2008-2018: A retrospective cohort study. Vaccine. 2019;37(37):5614-24. 10.1016/j.vaccine.2019.07.080 31402236

[24] World Health Organization (WHO). Monitoring the building blocks of health systems: a handbook of indicators and their measurement strategies. Geneva: WHO; 2010. Available from: https://iris.who.int/handle/10665/258734

[25] Essa-Hadad J Gorelik Y Vervoort J Jansen D Edelstein M . Understanding the health system barriers and enablers to childhood MMR and HPV vaccination among disadvantaged, minority or underserved populations in middle- and high-income countries: a systematic review. Eur J Public Health. 2024;34(2):368-74. 10.1093/eurpub/ckad232 38183166 PMC10990506

[26] Direction de la recherche, des études, de l’évaluation et des statistiques (DREES). La complémentaire santé. Acteurs, bénéficiaires, garanties. Édition 2024. [Supplementary health insurance. Stakeholders, beneficiaries, guarantees. Edition 2024]. Paris: DREES; 2024. French. Available from: https://drees.solidarites-sante.gouv.fr/publications-communique-de-presse/panoramas-de-la-drees/240710_Panorama_ComplementaireSante2024

[27] Direction de la recherche, des études, de l’évaluation et des statistiques (DREES). Non-recours aux prestations sociales : le manque d’information en tête des motifs selon les Français. [Non-use of social benefits: lack of information tops list of reasons according to the French]. Paris: DREES; 2022. Available from: https://drees.solidarites-sante.gouv.fr/jeux-de-donnees-communique-de-presse/non-recours-aux-prestations-sociales-le-manque-dinformation-en#:~:text=La%20quantification%20et%20l'identification,sup%C3%A9rieurs%20%C3%A0%2030%20%25%20en%20France

[28] Amrous N. Protection maternelle et infantile (PMI) : un recul de l’activité et une forte baisse des effectifs de médecins entre 2016 et 2019. [Maternal and child protection: a decline in activity and a sharp drop in the number of doctors between 2016 and 2019]. Report no. 1227. Paris: Direction de la recherche, des études, de l’évaluation et des statistiques; 2022. French. Available from: https://drees.solidarites-sante.gouv.fr/publications-communique-de-presse/etudes-et-resultats/protection-maternelle-et-infantile-pmi-un

[29] Litchfield I Shukla D Greenfield S . Impact of COVID-19 on the digital divide: a rapid review. BMJ Open. 2021;11(10):e053440. 10.1136/bmjopen-2021-053440 34642200 PMC8520586

[30] Dumesnil H Lutaud R Bellon-Curutchet J Deffontaines A Verger P . Dealing with the doctor shortage: a qualitative study exploring French general practitioners’ lived experiences, difficulties, and adaptive behaviours. Fam Pract. 2024;41(6):1039-47. 10.1093/fampra/cmae017 38521970

[31] World Health Organization Regional Office for Europe (WHO/Europe). Health and care workforce in Europe: time to act. Geneva: WHO/Europe; 2022. Available from: https://www.who.int/europe/publications/i/item/9789289058339

[32] Fasce A Schmid P Holford DL Bates L Gurevych I Lewandowsky S . A taxonomy of anti-vaccination arguments from a systematic literature review and text modelling. Nat Hum Behav. 2023;7(9):1462-80. 10.1038/s41562-023-01644-3 37460761

[33] Neufeind J Schmid-Küpke N Rehfuess E Betsch C Wichmann O . How a generally well-accepted measles vaccine mandate may lead to inequities and decreased vaccine uptake: a preregistered survey study in Germany. BMC Public Health. 2022;22(1):1846. 10.1186/s12889-022-14075-y 36192739 PMC9527387

[34] Pubert M, Giraud J, Pisarik J, Chaput H, Marbot C, Videau Y, et al. Prise en charge des patients en situation de vulnérabilité sociale : opinions et pratiques des médecins généralistes. [Care of patients in a situations of social vulnerability: opinions and practices of general practitioners]. Report no. 1089. Paris: Direction de la recherche, des études, de l’évaluation et des statistiques; 2018. French. Available from: https://drees.solidarites-sante.gouv.fr/publications/etudes-et-resultats/prise-en-charge-des-patients-en-situation-de-vulnerabilite-sociale

[35] Lendaro A, Rial-Sebbag E. Appropriations différentielles des normes sanitaires et des restrictions de libertés [Differential appropriations of health standards and restrictions of freedoms]. Report no. 21.40. Paris: Institut des Etudes et de la Recherche sur le Droit et la Justice; 2024. French. Available from: https://gip-ierdj.fr/fr/publications/normes-sanitaires-restrictions-libertes

[36] Lemaitre T Carrier N Farrands A Gosselin V Petit G Gagneur A . Impact of a vaccination promotion intervention using motivational interview techniques on long-term vaccine coverage: the PromoVac strategy. Hum Vaccin Immunother. 2019;15(3):732-9. 10.1080/21645515.2018.1549451 30457421 PMC6988881

[37] Verger P Cogordan C Fressard L Gosselin V Donato X Biferi M A postpartum intervention for vaccination promotion by midwives using motivational interviews reduces mothers’ vaccine hesitancy, south-eastern France, 2021 to 2022: a randomised controlled trial. Euro Surveill. 2023;28(38):2200819. 10.2807/1560-7917.ES.2023.28.38.2200819 37733238 PMC10515496

[38] Institut National de la Statistique et des Etudes Economiques (INSEE). Les revenus et le patrimoine des ménages. Niveau de vie et pauvreté par catégorie de communes. [Household income and wealth. Standard of living and poverty by category of municipality]. Montrouge: INSEE; 2024. French. Available from: https://www.insee.fr/fr/statistiques/7941413?sommaire=7941491

[39] Davin-Casalena B Jardin M Guerrera H J Mabille Tréhard H Lapalus D [The impact of the COVID-19 pandemic on first-line primary care in southeastern France: Feedback on the implementation of a real-time monitoring system based on regional health insurance data]. Rev Epidemiol Sante Publique. 2021;69(3):105-15. French. 33992499 10.1016/j.respe.2021.04.135 PMC8075812

