# Supplementary material for: Persistence of major socio-economic inequalities in childhood measles–mumps–rubella vaccination coverage and timeliness under vaccination mandates, France, 2015 to 2024
Source: Euro Surveill. 2025 Apr 24;30(16):2400674. doi: 10.2807/1560-7917.ES.2025.30.16.2400674 (PMC12023726; doi:10.2807/1560-7917.ES.2025.30.16.2400674)
Supplement: Supplement [file 24-00674_SCRONIAS_Supplement.pdf]

Supplementary material

This supplementary material is hosted by Eurosurveillance as supporting information alongside the article 'Persistence of major socioeconomic inequalities in childhood MMR vaccination coverage and timeliness under French vaccination mandates', on behalf of the authors, who remain responsible for the accuracy and appropriateness of the content. The same standards for ethics, copyright, attributions and permissions as for the article apply. Supplements are not edited by Eurosurveillance and the journal is not responsible for the maintenance of any links or email addresses provided therein.

Supplement 1. Sample inclusion hypotheses

Some vaccinations for which no reimbursement data exist, such as those from mother-child protection centres (MCPCs), cannot be retrieved from the national health data system. Failure to take this missing data into account may bias vaccination coverage (VC) estimates. To attenuate a potential bias, in accordance with the methodology of Santé publique France, the national public health agency, we excluded all children with no reimbursements for either a diphtheria, tetanus and poliomyelitis vaccination (before 2018) or a pentavalent/hexavalent (after 2018) vaccination during the 12 months after birth [1]. This hypothesis assumes children without these reimbursements were vaccinated in a MCPC and are likely to be vaccinated there again for the MMR vaccine. In all, 100,506/830,585 (12.7%) were excluded in 2015, 84,001/788,769 (10.6%) in 2017 and 72,293/751,066 (9.6%) in 2019.

Four départements (French administrative geographical entities) with high proportions of vaccines administered in MCPCs (Seine Saint-Denis, Haute-Saône, Mayotte, Guyane) were excluded by Santé publique France in their analyses. We chose to keep them after checking that excluding them yielded nearly identical results in VC, timeliness and delays at the national level.

Table S1 shows the proportion of subsidised social health insurance (SSHI) recipients among included and excluded children in each cohort. In all cohorts, excluded children were more likely than included children to be SSHI recipients and their proportion has increased over the years: from 32.4% of SSHI recipients in the 2015 cohort, to 43.3% in the 2019 cohort.

Reference

1. Lévy-Bruhl D, Fonteneau L, Vaux S, Barret AS, Antona D, Bonmarin I, et al. Assessment of the impact of the extension of vaccination mandates on vaccine coverage after 1 year, France, 2019. Euro Surveill. 2019 Jun;24(26). https://doi.org/10.2807/1560-7917.ES.2019.24.26.1900301

Table S1. Proportion of recipients of subsidised social health insurance (SSHI) per birth cohort and sample inclusion status, France (2015 N = 830,585; 2017 N = 788,769; 2019 N = 751,066)

	Non-recipients			SSHI recipients		
	N	Row %	Col %	N	Row %	Col %
2015 cohort						
Excluded[a] (MCPC)	71,294	67.6	11.4	34,212	32.4	16.6
Included	553,356	76.3	88.6	171,723	23.7	83.4
2017 cohort						
Excluded (MCPC)	52,914	63.0	9.1	31,087	37.0	15.2
Included	531,205	75.4	90.9	173,563	24.6	84.8
2019 cohort						
Excluded (MCPC)	41,020	56.7	7.6	31,273	43.3	14.9
Included	500,006	73.7	92.4	178,767	26.3	85.1

MCPC: Mother-child protection centre. SSHI: Subsidised social health insurance.
Data source: National health data system, March 2024

Supplement 2. Definition of delays and calculations

The first MMR dose, recommended at month 12, was considered late from the 14th month onward because the exact (with the day) delivery date was not available in the database. The same 2-month threshold was applied for the second MMR dose, recommended at month 18.

However, when the first dose occurred after month 18, we applied a three-month delay after this dose to calculate a delay for the second dose, because in this case, the second dose is recommended only one month after the first one to compensate for the delay in the first dose. That is, for a first dose at 24 months, delays for dose 2 were counted from month 27 onward (Figure S2).

Delays were thus calculated as the time in months between the dates that the pharmacy dispensed the vaccine by a pharmacy and the first month from which a vaccination was flagged as late. Delays were calculated for each dose among the children for whom that dose was dispensed late.

Figure S2. Delay calculation algorithm and examples

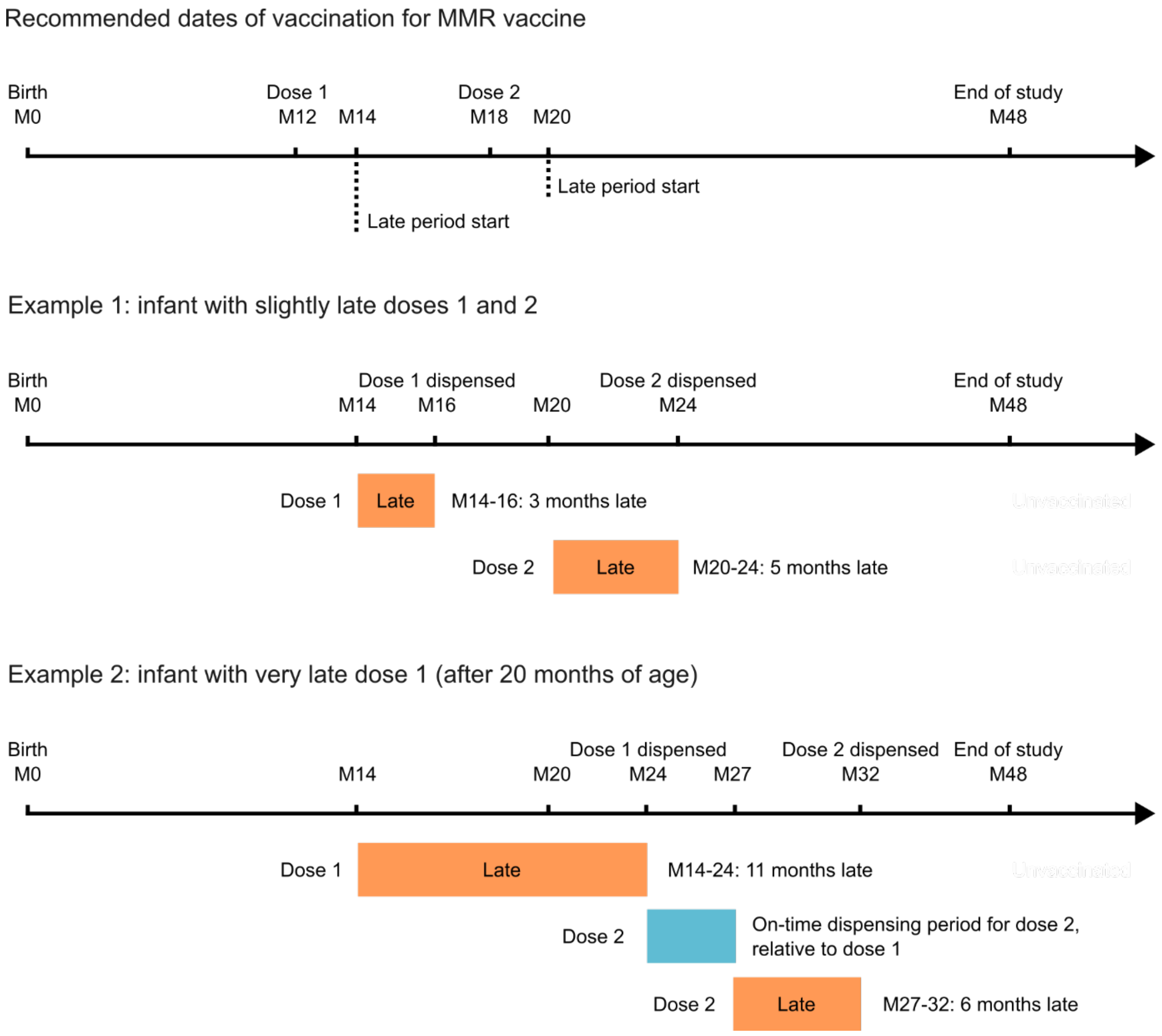

Supplement 3. Sensitivity analysis: VC and delay imputation for children in mother-child protection centres

Table S3. Sensitivity analysis: MMR vaccine dispensing status and delays for the 2019 cohort at 48 months with and without children vaccinated in mother-child protection centres, France

MMR dose and population	Type of analysis	Vaccine dispensing status at 48 months, first dose (%)	Vaccine dispensing status at 48 months, second dose (%)	Average cumulative delay[a] at 48 months (in months)
All infants	Main analysis[b], no imputed data	96.41	87.13	7.1
SSHI recipients		93.37	78.88	8.3
Not SSHI recipients		97.49	90.08	6.4
All infants	Sensitivity analysis[c], imputed data	96.56	87.89	6.9
SSHI recipients		94.06	81.28	8.0
Not SSHI recipients		97.53	90.45	6.2

MMR: measles, mumps and rubella. MCPC: mother-child protection centre.

[a] Time in months between the date a pharmacy dispensed the vaccine and the first month from which a vaccination was flagged as late. Delays were calculated for each dose among the children for whom that dose was dispensed late; children who had not received the dose of vaccine by 48 months were excluded from the calculation.
[b] Children born in 2019, alive four years after birth and for whom a pentavalent or hexavalent vaccination was dispensed during their first 12 months. This condition was applied to exclude children vaccinated in MCPCs. Exclusions: 72,293/751,066 (9.6%). Final number of children in the 2019 birth cohort, after these exclusions: N (2019) = 678,773.
[c] Full 2019 population size, without exclusions. To impute vaccine dispensing status, late dispensing and delays among children in MCPs, we made the following assumptions:

- 98% vaccination coverage for the first dose, and 95% vaccination coverage for the second dose;
- Late vaccinations among children vaccinated in MCPCs were half those observed among children vaccinated outside MCPCs;
- Mean delays among children vaccinated late in MCPCs were half those observed among children vaccinated outside MCPCs.

Data source: National health data system, March 2024

Supplement 4. Sensitivity analysis: delay threshold adjustment

Table S4. MMR vaccination coverage[a], percentages of children vaccinated late and mean delays at 48 months, among the three birth cohorts, according to two definitions of thresholds to define vaccination delays (France)

Variable	Denominator	Cohort birth year[b] 2015 N = 725,079	2017 N = 704,768	2019 N = 678,773
**Main analysis: delays start two months after the recommended vaccination date**				
Percent of:		%	%	%
1st MMR vaccine dispensed	All children	94.61	95.09	96.41
Late[c] 1st dose	Children with at least one dose dispensed	31.72	28.62	24.85
2nd MMR vaccine dispensed	All children	83.80	84.86	87.13
Late[c] 2nd dose	Children with both doses dispensed	33.37	26.16	20.37
Mean delay[d] of:		Months	Months	Months
The two cumulative doses[e]	Children with at least one dose dispensed, late first and/or second doses	8.2	7.3	7.1
**Sensitivity analysis: delays start one month after the recommended vaccination date**				
Percent of:		%	%	%
1st MMR vaccine dispensed	All children	94.61	95.09	96.41
Late[f] 1st dose	Children with at least one dose dispensed	51.47	50.18	46.42
2nd MMR vaccine dispensed	All children	83.80	84.86	87.13
Late[f] 2nd dose	Children with both doses dispensed	41.37	34.64	29.08
Mean delay[d] of:		Months	Months	Months
The two cumulative dose[e]	Children with at least one dose dispensed, late first and/or second doses	7.3	6.1	5.6

MMR: measles, mumps and rubella.

[a] The dates that pharmacies dispensed the MMR vaccines were used as proxies for the injection date to estimate vaccination coverage rates.

[b] This study included only children born in 2015, 2017 and 2019, who were alive four years after birth and who had a DTaP vaccine dispensed at least once (before 2018) or a pentavalent or hexavalent vaccine dispensed at least once (2018 onwards) by their first birthday: this condition was applied to exclude children vaccinated in MCPCs [5]. Final numbers of children in the birth cohorts, after these exclusions: N (2015) = 725,079; N (2017) = 704,768; N (2019) = 678,773.

[c] MMR vaccine doses were considered dispensed late from month 14 for the first dose and month 20 for the second dose onward. See Supplement 2 for the handling of specific cases, such as a first dose dispensed after the mandatory date for the second.

[d] Children without an MMR vaccine dispensed by 48 months were excluded from the delay calculation.

[e] Mean cumulative delay includes children whose first and/or second MMR dose was dispensed late.

[f] MMR vaccine doses were considered dispensed late from month 13 for the first dose and month 19 for the second dose onward. See Supplement 2 for the handling of specific cases, such as a first dose dispensed after the mandatory date for the second.

Data source: National health data system, March 2024
